# Clinical and microscopic predictors of *Entamoeba histolytica* intestinal infection in travelers and migrants diagnosed with *Entamoeba histolytica/dispar* infection

**DOI:** 10.1371/journal.pntd.0006892

**Published:** 2018-10-29

**Authors:** Steven Van Den Broucke, Jacob Verschueren, Marjan Van Esbroeck, Emmanuel Bottieau, Jef Van den Ende

**Affiliations:** 1 Department of Clinical Sciences, Institute of Tropical Medicine, Antwerp, Belgium; 2 Faculty of Medicine and Health Sciences, University Antwerp, Belgium; National University of Singapore, SINGAPORE

## Abstract

**Background:**

Amebiasis is a protozoal infection caused by *Entamoeba histolytica*, while the morphologically indistinguishable *E*. *dispar* is considered as non-pathogenic. Polymerase chain reaction (PCR) assays are necessary to differentiate both species. The most common clinical presentations of *E*. *histolytica* disease are amebic colitis and amebic liver abscess, but asymptomatic infection is also possible. We assessed the frequency and pattern of clinical symptoms and microscopic features in travelers/migrants associated with *E*. *histolytica* intestinal infection and compared them to those found in individuals with *E*. *dispar* infection.

**Methods:**

We conducted a retrospective study at the travel clinic of the Institute of Tropical Medicine, Antwerp, Belgium on travelers/migrants found from 2006 to 2016 positive for *Entamoeba histolytica/dispar* through antigen detection and/or through microscopy confirmed by PCR. All files of individuals with a positive PCR for *E*. *histolytica* (= cases) and a random selection of an equal number of *Entamoeba dispar* carriers (= controls) were reviewed. We calculated the sensitivity, specificity and likelihood ratios (LRs) of clinical symptoms (blood in stool, mucus in stool, watery diarrhea, abdominal cramps, fever or any of these 5 symptoms) and of microscopic features (presence of trophozoites in direct and in sodium acetate-acetic acid-formalin (SAF)-fixed stool smears) to discriminate between *E*. *histolytica* and *E*. *dispar* infection.

**Results:**

Of all stool samples positive for *Entamoeba histolytica/dispar* for which PCR was performed (n = 810), 30 (3.7%) were true *E*. *histolytica* infections, of which 39% were asymptomatic. Sensitivity, specificity and positive LRs were 30%, 100% and 300 (p 0.007) for presence of blood in stool; 22%, 100% and 222 (p 0.03) for mucus in stool; 44%, 90% and 4.7 (p 0.009) for cramps and 14%, 97% and 4.8 (p = 0.02) for trophozoites in direct smears. For watery diarrhea, fever and for trophozoites in SAF fixated smears results were non-significant.

**Conclusions:**

*E*. *histolytica* infection was demonstrated in a small proportion of travelers/migrants with evidence of *Entamoeba histolytica/dispar* infection. In this group, history of blood and mucus in stool and cramps had good to strong confirming power (LR+) for actual *E*. *histolytica* infection. Trophozoites were also predictive for true *E*. *histolytica* infection but in direct smears only.

## Author summary

In the present work, we found that *E*. *histolytica* intestinal infections are rarely diagnosed among travelers and migrants presenting in a national reference travel clinic in Europe. Microscopic finding of cysts or trophozoites and antigen testing cannot discriminate between *Entamoeba histolytica/dispar* infection, which leads to overdiagnosis of *E*. *histolytica* infections in low resource settings where PCR is not available. We found visualization of trophozoites under the microscope helpful in discriminating between *E*. *histolytica* and *E*. *dispar* infection in direct smears. Hematophagy is a very rare finding but in our experience was always associated with *E*. *histolytica* infection. In a context where only microscopy is available, a patient presenting with blood or mucus in stool or cramps should anyhow be treated as amoebiasis if *Entamoeba histolytica/dispar* cysts/trophozoites are found. Nevertheless it is worth noting that a sizeable proportion of *E*. *histolytica* cases were asymptomatic. Last, our study suggests that *E*. *dispar* might be pathogenic but symptoms in *E*. *histolytica* infected patients were clearly more often suggestive of intestinal tissue invasion.

## Introduction

Amebiasis is a protozoal infection caused by *Entamoeba histolytica*. The most common clinical presentations of disease are amebic colitis and amebic liver abscess. Before molecular tests allowed distinction between *Entamoeba* species[1]^,^[2], the estimations of the worldwide burden of amoebiasis indicated that approximately 500 million people were infected by *E*. *histolytica*, and 10% of these individuals had invasive amoebiasis. Moreover, it was estimated that 100,000 patients per year died due to the clinical complications of the disease[3]. The genus *Entamoeba* contains many species of which *Entamoeba histolytica*, *Entamoeba dispar*, *Entamoeba coli*, *Entamoeba hartmanni*, and to a much lesser extent *Entamoeba moshkovskii* and *Entamoeba polecki*, are found in the human intestinal tract. Cysts of *E*. *histolytica*, *E*. *dispar*, and *E*. *moshkovskii* are morphologically indistinguishable[4]^,^[5]^,^[6] but the species are biochemically and genetically different[7]. Towards the end of the 20^th^ century, Polymerase Chain Reaction (PCR)-assays that allowed to differentiate between *E*. *histolytica* and *E*. *dispar* infection led to a re-assessment of the disease burden and indicate that earlier reports had largely overestimated the true number of *E*. *histolytica* infections. More recent reports showed in addition varied frequencies of asymptomatic *E*. *histolytica* carriage in different populations, ranging from 0–2% in South-Africa and Ivory Coast to 21% in Egypt, with intermediate prevalence of 13.8% reported in rural Mexico and 9.6% in Vietnam[8]^,^[9]^,^[10]^,^[11]. In studies dating from before PCR could discriminate between *E*. *histolytica* and *E*. *dispar* infection, a 4% prevalence of asymptomatic *E*. *histolytica/dispar* infection was found in travelers returning from the tropics[12]. Notwithstanding, the ratio of symptomatic vs asymptomatic *E*. *histolytica* infections remains largely unknown. Though *E*. *dispar* is considered non-pathogenic, it has been reported that *E*. *dispar* may be the causative agent of intestinal and extra-intestinal symptoms in humans[13]^,^[14].

The finding of trophozoites (or vegetative forms) in fresh stool samples is generally considered predictive of true *E*. *histolytica* infection, especially when large trophozoites containing red blood cells are found (hematophagy)[15]^,^[16]^,^[17], but it is not known whether the presence of trophozoites found after fixation of stools differs between *E*. *histolytica* and *E*. *dispar*.

In the present work, we aimed to determine the frequency of *E*. *histolytica* infection among travelers and migrants presenting with an *Entamoeba histolytica/dispar* infection diagnosed by microcopy and/or antigen detection at the travel clinic of the Institute of Tropical Medicine of Antwerp, Belgium. In addition, we assessed the predictive value of microscopic features and clinical symptoms for *E*. *histolytica* intestinal infection in this study group and correlated the finding of trophozoites in fresh and fixed stool samples with species identification.

## Materials and methods

### Study setting and population

The Institute of Tropical Medicine, Antwerp (ITMA) is the national reference clinic for tropical medicine in Belgium, with on average about 6500 consultations a year for post-travel care. For this retrospective study, all files of symptomatic and asymptomatic individuals having attended the travel clinic of the ITMA from May 2006 to March 2016 and positive for *Entamoeba histolytica/dispar* through antigen detection and/or through microscopy (trophozoites or cysts) confirmed by PCR, were retrieved. The medical records of all travelers and migrants proven to be infected with *E*. *histolytica* during the study period were then reviewed. An equal number of files of patients with confirmed E. *dispar* intestinal infection were randomly chosen and analyzed for a case control comparison.

### Epidemiological and clinical data

Relevant clinical and laboratory data were extracted, de-identified and entered in a Microsoft Access 2010 database. Variables included: demographic data including country of origin, month and year of first *Entamoeba* positive test, most recent travel destination and, for the symptomatic included cases and controls, the following clinical features at presentation: blood in stool, mucus in stool, watery diarrhea, abdominal cramps and fever, as reported in the medical files.

### Laboratory workup

All stool samples were analyzed by microscopic examination of direct smears and wet mounts after formalin-ether concentration (Loughlin and Spitz, 1949[18]). A limited number of samples with high suspicion for amebic dysentery was urgently sent to the lab for immediate examination. In case a fresh stool sample could not be produced in ITMA, the patient received a package to collect stools at home and instructions to mix part of the stools immediately with a sodium acetate-acetic acid-formalin (SAF) solution. Both fixed and unfixed portions were sent to ITMA for examination. In case the stool sample was produced at ITMA, part of it was mixed with SAF-solution within 20 minutes on request by the treating physician. All SAF-fixed stool samples were examined by microscopy after iron hematoxylin Kinyoun staining. Antigen detection with the enzyme-linked immunosorbent assay (ELISA) E. histolytica ProSpecT ELISA Microplate assay (Remel, Lenexa, Kansas, USA), was performed when requested by the treating physician. Since microscopic distinction of *E*. *histolytica*, *E*. *dispar* and some other *Entamoeba* species is not possible, an *E*. *histolytica* and *E*. *dispar* specific real-time PCR (Cnops and Van Esbroeck, 2010[19]) was performed on all samples positive by microscopy and/or antigen detection. Direct smears were examined for the presence of hematophagy. In SAF-fixed stool this feature cannot be used, given possible superposition of erythrocytes over parasites, instead of within parasites.

### Predictors of *E*. *histolytica* infection in *E*. *histolytica/dispar* positive individuals

Among individuals found with *E*. *histolytica/dispar* intestinal infection, we analyzed the respective frequencies of the presence of *E*. *histolytica* and *E*. *dispar* trophozoites and cysts as well as the pattern of clinical findings (blood and/or mucus in stool, watery diarrhea, presence of abdominal cramps, fever or any symptom). Sensitivity, specificity and likelihood ratios (LRs) were calculated, using the PCR as reference diagnostic standard. Finally, we assessed whether hematophagy can be used as a criterium to distinguish *E*. *histolytica* and *E*. *dispar* species in direct stool smears.

### Statistical analysis

Laboratory test results were stored in the Laboratory Information System AS/400 (IBM, USA). Data mining was performed with the SAP Business Objects (SAP, USA) program. Statistical analyses were done with Epi-Info (CDC 2015). Dichotomic variables where compared with Fisher exact test, minimum significance p<0.05.

### Ethics statement

This was a retrospective analysis of data collected during clinical care over an 11-year period. Ethical clearance was obtained from the institutional review board at ITMA. Laboratory queries were obtained in an anonymous way. Clinical data were then retrieved through an encoded link and de-identified for analysis according to the Belgian legislation.

## Results

### Dataset

From May 2006 till March 2016 parasitological examination was performed on 40,638 stool samples. Of these 868 (2.1%) were found positive for *Entamoeba histolytica/dispar* through antigen detection and/or through microscopy confirmed by PCR. After removing results of follow-up samples, *E*. *histolytica* was detected in 30/826 samples: 3.6% of all stool samples positive for *E*. *histolytica/dispar* and 0.07% of all examined stool samples. *E*. *dispar* was detected in 714 (86.4%) samples, neither *E*. *histolytica* nor *E*. *dispar* in 50, and PCR was technically not feasible in 16 because no fresh stool sample was received. No co-infections with *E*. *histolytica* and *E*. *dispar* were found.

Antigen detection was performed in 396 of the 744 samples with *E*. *histolytica* or E. *dispar* as confirmed by PCR. In 16 samples, the antigen test was positive, with negative PCR for *E*. *histolytica* or *E*. *dispar* and negative microscopy (or microscopy not done), while in 1 *E*. *histolytica* PCR-confirmed patient antigen testing was positive with negative microscopy. The antigen test was positive in 15/16 (94%) *E*. *histolytica* positive and 275/380 (72%) *E*. *dispar* positive samples.

### Clinical predictors

#### Baseline clinical characteristics

*E*. *histolytica* cases (n = 30) were evenly distributed throughout the study period with no cluster phenomenon. Mean age was 36.8 years (range 4–80 years) and 21 (70%) samples were from males (Table 1). Regions of most recent travels were Africa (20 cases, 69%), Asia (8 cases, 24%), Latin-America (1 case, 3%) and Europe (1 case, 3%).

**Table 1:** Baseline comparison patients with E histolytica and controls with E dispar.

	E. histolytica (n = 30)	E. dispar (n = 30)	Significance
Gender M/F	21/9	24/6	Ns
Age (mean)	36.8	42.8	Ns
Africa/all	20/30	18/30	Ns

Of the 30 randomly selected *E*. *dispar* cases, mean age was 43 years (range 23–72 years) and 24 (80%) were males. Most recent travel regions were Africa (18 cases, 60%), Asia (3 cases, 10%), Latin-America (1 case, 3%) and Europe (8 cases, 27%).

Gender, age and travel destination were not significantly different between the *E*. *histolytica* and *E*. *dispar* cases.

Two of the patients with *E*. *histolytica* in the stool had an amebic liver abscess. One had recto-colitis with rectal prolapse.

#### Clinical predictors of E. histolytica vs. dispar

In order to exclude pathogenicity by coinfections, clinical predictors were evaluated only on mono-infections with *E*. *histolytica* or *E*. *dispar*. Four patients with *E*. *dispar* infection were co-infected with *Giardia intestinalis* (n = 2), *Strongyloides stercoralis* (n = 1) or *Schistosoma mansoni* (n = 1). Eight *E*. *histolytica* patients were co-infected with one or two of the following: *Giardia intestinalis* (n = 5), *Trichuris trichiura* (n = 2), *Ankylostoma duodenale* (n = 1), *Schistosoma mansoni* (n = 1) *and/or Campylobacter* (n = 1).

Overall, sensitivity of the different symptoms was low (Table 2). About 40% of *E*. *histolytica* infections were fully asymptomatic. Bloody stools, mucus and abdominal cramps were significantly correlated with *E*. *histolytica*, with a specificity of resp. 100, 100 and 90%. The presence of “any symptom” was not predictive for *E*. *histolytica* infection.

**Table 2:** Case control study: Prediction of presence of E. histolytica by clinical data in patients with mono-infections with stool microscopy positive for E. histolytica/E. dispar.

	*E*. *histolytica*	*E*. *dispar*					
	N/total	N/total	Sensitivity	Specificity	LR+	LR-	P-value
**Blood in stool**	6/20	0/22	30	100	300*	0.7	0.007
**Mucus in stool**	4/18	0/22	22	100	222*	0.8	0.030
**Cramps**	8/18	2/21	44	90	4.7	0.6	0.009
**Watery diarrhea**	6/19	11/22	32	50	0.6	1.4	0.2
**Fever**	2/20	3/22	10	86	0.7	1.0	0.6
**Any Symptom**	13/21	12/22	62	45	1.1	0.8	0.420

LR+: Positive likelihood ratio. LR-: Negative likelihood ratio.

* Division by 99.9 instead of 100.

### Laboratory predictors

When only examination of direct smears was considered, the finding of trophozoites was predictive of E. histolytica (p = 0.02), although sensitivity was very low (14%) (Table 3)

**Table 3:** Crosstab of finding of trophozoites for identification of E histolytica in direct smears.

	E. histolytica		E. dispar			LR
Trophozoite	3			17	20	
		14%	3%			4.82
		86%	97%			0.88
Cyst	18			557	575	
Total	21			574	595	

In contrast, the finding of trophozoites in fixed samples was not predictive of E. histolytica (p = 0.2; Table 4).

**Table 4:** Crosstab of finding of trophozoites for identification of E histolytica in fixed samples.

	E. histolytica		E. dispar			LR
Trophozoite	14			250	264	
		74%	63%			1.17
		26%	37%			0.71
Cyst	5			146	151	
Total	19			396	415	

#### Hematophagy

In 5 immediately examined fresh stool (n = 3) and rectal pus samples (n = 2) hematophagous trophozoites were found, all of which were confirmed as *E*. *histolytica*. All 5 patients presented with bloody diarrhea, but co-infection was detected in 3 (1 with *Campylobacter jejuni* and 2 with *Giardia intestinalis)*.

## Discussion

### Findings

In our Belgian reference clinic for tropical medicine we identified 3.6% (30/826) of *Entamoeba histolytica/dispar* infections as true *E*. *histolytica* infections by PCR. This confirms the finding in other studies[20]^,^[21] that the bulk of *Entamoeba histolytica/dispar* infections are caused by *E*. *dispar* amoeba. True *E*. *histolytica* enteritis is a rare finding in patients presenting in our reference center, with on average less than 3 cases detected per year.

In our study, the presence of blood or mucus in stool or abdominal cramps are clearly significant predictors (p < 0.005) of true *E*. *histolytica* infections in case *Entamoeba histolytica/dispar* cysts or trophozoites were found on microscopy. Likelihood ratios of symptoms can be used similarly to test results to calculate the probability of disease according to the Bayes theorem. Since the positive LR is the ratio between true positive and false positive rates, a symptom, even if infrequent in a given disease, can have a high LR+ (a high confirming power) if it is rarer in the competing[22]. Indeed we observed that blood or mucus in stool or abdominal cramps were not that frequent in true *E*. *histolytica* infections, but that these symptoms were almost never present in the matched patients with *E*. *dispar*, which explains the high LR+. Therefore, in a context where only microscopy is available, a patient presenting with blood or mucus in stool or cramps should anyhow be treated as amoebiasis if *Entamoeba histolytica/dispar* cysts/trophozoites are found. Nevertheless it is also worth noting that a sizeable proportion of *E*. *histolytica* cases were asymptomatic. Relying only on one of the three clinical predictors would have missed 10 true *E*. *histolytica* infections in our cohort.

Hematophagy is considered a discriminative microscopic criterion to distinguish *E*. *histolytica* from *E*. *dispar* infection[15]^,^[16]^,^[17]. This was also demonstrated in this study in which 5/5 hematophagous trophozoites found in immediately examined samples proved to be *E*. *histolytica*.

Finding trophozoites in direct smears had a LR+ for *E*. *histolytica* of 4.8, corresponding to a good confirming power. However, the LR- of 0.9 indicated that the absence of trophozoites, did not rule out *E*. *histolytica* infection. The non-significant LR+ of 1.2 for trophozoites in SAF fixed stool samples confirmed that this method cannot be used for species prediction.

The non-pathogenicity of *Entamoeba dispar* is questioned by several authors[23],[14]. A study by Ximénez and colleagues suggests the existence of several different genotypes of *E*. *dispar* that can be associated to, or be potentiality responsible for, intestinal or liver tissue damage, similar to that observed with *E*. *histolytica*[13]. The difference in percentage of patients presenting with any symptom in patients with mono-infections with *E*. *histolytica* vs *E*. *dispar* was not significant (61% vs 55%, p value 0.42). This is not equivalent to stating that all symptoms of the 55% patients with symptomatic *E*. *dispar* infections were attributable to the *E*. *dispar* amoebae. Our study was not designed to show a pathogenic effect of *E*. *dispar*. However, the high frequency of symptoms in patients with *E*. *dispar* mono-infection supports Ximénez’s hypothesis, but symptoms in *E*. *histolytica* infected patients were clearly more often suggestive of intestinal tissue invasion.

### Limitations

Our study has several limitations. It was a single-center study and the total number of *E*. *histolytica* infections found might not be representative for all returning travelers. In patients consulting at our center, we found 30 *E*. *histolytica* infections over 10 years, whereas the total number of *E*. *histolytica* infections diagnosed in our laboratory receiving stool samples from all over Belgium was 124 over the same period. Next, it was a retrospective study meaning that collection of data was not systematic. However, given the low number of confirmed *E*. *histolytica* infections in the 810 samples tested by PCR, the impact of missing analyses is likely marginal. In 50 samples positive by microscopy PCR was negative for both *E*. *histolytica* and *E*. *dispar* which probably indicates incorrect identification as infections with species such as *E*. *moshkovskii and E*. *polecki* are considered to be rare. A difference in clinical presentation in patients with *E*. *histolytica* and *E*. *dispar* infection is a possible confounding factor since clinicians might have asked less stool samples in asymptomatic patients. This might have underestimated the true prevalence of these infections. Nevertheless, the proportion of asymptomatic patients in our case-control group did not differ significantly. Furthermore, requesting stool analysis including antigen testing was clinician driven and an unknown number of *E*. *histolytica/dispar* infections may have been missed, in particular in asymptomatic travelers. The most trustworthy method to detect all *E*. *histolytica* and *E*. *dispar* infections, would have been to perform PCR on all stool samples of all symptomatic and asymptomatic travelers[7]^,^[24]. During the study period, this method was not part of common practice, though this may change with the deployment of multiplex PCR platforms to analyze stool samples. Last, quantification of pathogens is usually linked with disease severity, which is mostly demonstrated for bacterial diseases[25]. We opted however to correlate our symptoms to the qualitative and not the quantitative interpretation of the PCR results because the goal of our study was identification of *E*. *histolytica* as such–which is treated even in asymptomatic patients–and not determination of pathogenicity.

## Conclusion

In conclusion, even in a national reference travel clinic in Europe, *E*. *histolytica* intestinal infections are rarely diagnosed. Finding trophozoites is helpful in discriminating between *E*. *histolytica* and *E*. *dispar* infection in direct smears but not in SAF fixed samples. History of blood and mucus in stool and cramps in individuals with microscopic evidence of *E*. *histolytica/dispar* infection had good to strong predictive weights for actual *E*. *histolytica* infection. Hematophagy was a very rare finding but in our experience was always associated, when requested, with *E*. *histolytica* infection. Our study suggests that *E*. *dispar* might be pathogenic but symptoms in *E*. *histolytica* infected patients were clearly more often suggestive of intestinal tissue invasion.

## Supporting information

S1 Checklist STROBE checklist. (DOC)
