# Supplementary material for: Clinical and microscopic predictors of Entamoeba histolytica intestinal infection in travelers and migrants diagnosed with Entamoeba histolytica/dispar infection
Source: PLoS Negl Trop Dis. 2018 Oct 29;12(10):e0006892. doi: 10.1371/journal.pntd.0006892 (PMC6233926; doi:10.1371/journal.pntd.0006892)
Supplement: S1 Checklist — (DOC) [file pntd.0006892.s001.doc]

STROBE Statement—checklist of items that should be included in reports of observational studies

|  | Item No | Recommendation |
| --- | --- | --- |
| **Title and abstract** | 1 | (*a*) Indicate the study’s design with a commonly used term in the title or the abstract  Clinical and microscopic predictors of Entamoeba histolytica intestinal infection in travelers and migrants diagnosed with *Entamoeba histolytica/dispar* infection |
|  |  | (*b*) Provide in the abstract an informative and balanced summary of what was done and what was found Background Amebiasis is a protozoal infection caused by *Entamoeba histolytica,* while the morphologically indistinguishable *E. dispar* is considered as non-pathogenic. Polymerase chain reaction (PCR) assays are necessary to differentiate both species. The most common clinical presentations of *E. histolytica* disease are amebic colitis and amebic liver abscess, but asymptomatic infection is also possible. We assessed the frequency and pattern of clinical symptoms and microscopic features in travelers/migrants associated with *E. histolytica* intestinal infection and compared them to those found in individuals with *E. dispar* infection. Methods We conducted a retrospective study at the travel clinic of the Institute of Tropical Medicine, Antwerp, Belgium on travelers/migrants found from 2006 to 2016 positive for *Entamoeba histolytica/dispar* through antigen detection and/or through microscopy confirmed by PCR. All files of individuals with a positive PCR for *E. histolytica* (= cases) and a random selection of an equal number of *Entamoeba dispar* carriers (= controls) were reviewed. We calculated the sensitivity, specificity and likelihood ratios (LRs) of clinical symptoms (blood in stool, mucus in stool, watery diarrhea, abdominal cramps, fever or any of these 5 symptoms) and of microscopic features (presence of trophozoites in direct and in sodium acetate-acetic acid-formalin (SAF)-fixed stool smears) to discriminate between *E. histolytica* and *E. dispar* infection. Results Of all stool samples positive for *Entamoeba histolytica/dispar* for which PCR was performed (n=810), 30 (3.7%) were true *E. histolytica* infections. Sensitivity, specificity and positive LRs were 30%, 100% and 300 (p 0.007) for presence of blood in stool; 22%, 100% and 222 (p 0.03) for mucus in stool; 44%, 90% and 4.7 (p 0.009) for cramps and 14%, 97% and 4.8 (p=0.02) for trophozoites in direct smears. For watery diarrhea, fever and for trophozoites in SAF fixated smears results were non-significant. Conclusions *E. histolytica* infection was demonstrated in a small proportion of travelers/migrants with evidence of *Entamoeba histolytica/dispar* infection. In this group, history of blood and mucus in stool and cramps had good to strong confirming power (LR+) for actual *E. histolytica* infection. Trophozoites were also predictive for true *E. histolytica* infection but in direct smears only. |
| Introduction | | |
| Background/rationale | 2 | Explain the scientific background and rationale for the investigation being reported  Amebiasis is a protozoal infection caused by *Entamoeba histolytica*. The most common clinical presentations of disease are amebic colitis and amebic liver abscess. Before molecular tests allowed distinction between *Entamoeba* species^1^*,*^2^, the estimations of the worldwide burden of amoebiasis indicated that approximately 500 million people were infected by *E.* *histolytica*, and 10% of these individuals had invasive amoebiasis. Moreover, it was estimated that 100,000 patients per year died due to the clinical complications of the disease^3^. The genus Entamoeba contains many species of which Entamoeba histolytica, Entamoeba dispar, Entamoeba coli, Entamoeba hartmanni, and to a much lesser extent Entamoeba moshkovskii and Entamoeba polecki, are found in the human intestinal tract. Cysts of E. histolytica, E. dispar, and E. moshkovskii are morphologically indistinguishable^4^*,*^5^*,*^6^ but the species are biochemically and genetically different^7^. Towards the end of the 20^th^ century, Polymerase Chain Reaction (PCR)-assays that allowed to differentiate between *E. histolytica*  and *E. dispar* infection led to a re-assessment of the disease burden and indicate that earlier reports had largely overestimated the true number of *E. histolytica* infections. More recent reports showed in addition varied frequencies of asymptomatic *E. histolytica* carriage in different populations, ranging from 0-2% in South-Africa and Ivory Coast to 21% in Egypt, with intermediate prevalence of 13.8% reported in rural Mexico and 9.6% in Vietnam^8^*,*^9^*,*^10^*,*^11^. In studies dating from before PCR could discriminate between *E. histolytica* and *E. dispar* infection, a 4% prevalence of asymptomatic *E. histolytica/dispar* infection was found in travelers returning from the tropics^12^. Notwithstanding, the ratio of symptomatic vs asymptomatic *E. histolytica*  infections remains largely unknown. Though *E. dispar* is considered non-pathogenic, it has been reported that *E. dispar* may be the causative agent of intestinal and extra-intestinal symptoms in humans^14,15^. The finding of trophozoites (or vegetative forms) in fresh stool samples is generally considered predictive of true *E. histolytica* infection, especially when large trophozoites containing red blood cells are found (hematophagy)^16,17,18^, but it is not known whether the presence of trophozoites found after fixation of stools differs between *E. histolytica* and *E. dispar*. |
| Objectives | 3 | State specific objectives, including any prespecified hypotheses  In the present work, we aimed to determine the frequency of *E. histolytica*  infection among travelers and migrants presenting with an *Entamoeba histolytica/dispar* infection diagnosed by microcopy and/or antigen detection at the travel clinic of the Institute of Tropical Medicine of Antwerp, Belgium. In addition, we assessed the predictive value of microscopic features and clinical symptoms for *E. histolytica* intestinal infection in this study group and correlated the finding of trophozoites in fresh and fixed stool samples with species identification. |
| Methods | | |
| Study design | 4 | Present key elements of study design early in the paper  For this **retrospective** study, all files of symptomatic and asymptomatic individuals having attended the travel clinic of the ITMA from May 2006 to March 2016 and positive for *Entamoeba histolytica/dispar* through antigen detection and/or through microscopy (trophozoites or cysts) confirmed by PCR, were retrieved. The medical records of **all travelers and migrants proven to be infected with *E. histolytica* during the study period** were then reviewed. An equal number of files of patients with confirmed E*. dispar* intestinal infection were randomly chosen and analyzed for a **case control comparison**. |
| Setting | 5 | Describe the setting, locations, and relevant dates, including periods of recruitment, exposure, follow-up, and data collection  The **Institute of Tropical Medicine, Antwerp (ITMA)** is the national reference clinic for tropical medicine in Belgium, with on average about 6500 consultations a year for post-travel care. For this retrospective study, all files of symptomatic and asymptomatic individuals having attended the travel clinic of the ITMA **from May 2006 to March 2016** and positive for *Entamoeba histolytica/dispar* through antigen detection and/or through microscopy (trophozoites or cysts) confirmed by PCR, were retrieved. The medical records of all travelers and migrants proven to be infected with *E. histolytica* during the study period were then reviewed. An equal number of files of patients with confirmed E*. dispar* intestinal infection were randomly chosen and analyzed for a case control comparison. Epidemiological and clinical data Relevant clinical and laboratory data were extracted, de-identified and entered in a Microsoft Access 2010 database. **Variables included:** demographic data including country of origin, month and year of first *Entamoeba* positive test, most recent travel destination and, for the symptomatic included cases and controls, the following clinical features at presentation: blood in stool, mucus in stool, watery diarrhea, abdominal cramps and fever, as reported in the medical files.  PCR (Cnops and Van Esbroeck, 2010^20^) was performed on all samples positive by microscopy and/or antigen detection. |
| Participants | 6 | (*a*) *Cohort study*—Give the eligibility criteria, and the sources and methods of selection of participants. Describe methods of follow-up  All files off symptomatic and asymptomatic individuals having attended the travel clinic of the ITMA from May 2006 to March 2016 and positive for *Entamoeba histolytica/dispar* through antigen detection and/or through microscopy (trophozoites or cysts) confirmed by PCR, were retrieved  **Diagnostic methods**: All stool samples were analyzed by microscopic examination of direct smears and wet mounts after formalin-ether concentration (Loughlin and Spitz, 1949^19^). A limited number of samples with high suspicion for amebic dysentery was urgently sent to the lab for immediate examination. In case a fresh stool sample could not be produced in ITMA, the patient received a package to collect stools at home and instructions to mix part of the stools immediately with a sodium acetate-acetic acid-formalin (SAF) solution. Both fixed and unfixed portions were sent to ITMA for examination. In case the stool sample was produced at ITMA, part of it was mixed with SAF-solution within 20 minutes on request by the treating physician. All SAF-fixed stool samples were examined by microscopy after iron hematoxylin Kinyoun staining. Antigen detection with the enzyme-linked immunosorbent assay (ELISA) E. histolytica ProSpecT ELISA Microplate assay (Remel, Lenexa, Kansas, USA), was performed when requested by the treating physician. Since microscopic distinction of *E. histolytica*, *E. dispar* and some other *Entamoeba* species is not possible, an *E. histolytica* and *E. dispar* specific real-time PCR (Cnops and Van Esbroeck, 2010^20^) was performed on all samples positive by microscopy and/or antigen detection. Direct smears were examined for the presence of hematophagy. In SAF-fixed stool this feature cannot be used, given possible superposition of erythrocytes over parasites, instead of within parasites.  *Case-control study*—Give the eligibility criteria, and the sources and methods of case ascertainment and control selection. Give the rationale for the choice of cases and controls An equal number of files of patients with confirmed E*. dispar* intestinal infection were randomly chosen and analyzed for a case control comparison  *Cross-sectional study*—Give the eligibility criteria, and the sources and methods of selection of participants |
|  |  | (*b*) *Cohort study*—For matched studies, give matching criteria and number of exposed and unexposed  *Case-control study*—For matched studies, give matching criteria and the number of controls per case  An equal number of files of patients with confirmed E*. dispar* intestinal infection were randomly chosen and analyzed for a case control comparison. We found 30 *E.histolytica* infected patients, thus 30 files of *E. dispar* infected patients were retrieved |
| Variables | 7 | Clearly define all outcomes, exposures, predictors, potential confounders, and effect modifiers. Give diagnostic criteria, if applicable  Variables included: demographic data including country of origin, month and year of first *Entamoeba* positive test, most recent travel destination and, for the symptomatic included cases and controls, the following clinical features at presentation: blood in stool, mucus in stool, watery diarrhea, abdominal cramps and fever, as reported in the medical files. **Predictors of *E. histolytica* infection in *E. histolytica/dispar* positive individuals**  Among individuals found with *E. histolytica/dispar* intestinal infection, we analyzed the respective frequencies of the presence of *E. histolytica* and *E. dispar* trophozoites and cysts as well as the pattern of clinical findings (blood and/or mucus in stool, watery diarrhea, presence of abdominal cramps, fever or any symptom).  We assessed whether hematophagy can be used as a criterium to distinguish *E. histolytica* and *E. dispar* species in direct stool smears.  **Potential confounders:** A difference in clinical presentation in patients with *E. histolytica* and *E. dispar* infection is a possible confounding factor since clinicians might have asked less stool samples in asymptomatic patients. |
| Data sources/ measurement | 8* | For each variable of interest, give sources of data and details of methods of assessment (measurement). Describe comparability of assessment methods if there is more than one group  The medical records of all travelers and migrants proven to be infected with *E. histolytica* during the study period were then reviewed  Laboratory test results were stored in the Laboratory Information System AS/400 (IBM, USA) |
| Bias | 9 | Describe any efforts to address potential sources of bias  The *E. dispar* case-control group was chosen randomly, limiting possible bias. Baseline comparison between *E. histolytica* and *E. dispar* infected patients did not differ significantly.  In order to exclude pathogenicity by coinfections, clinical predictors were evaluated only on mono-infections with *E. histolytica* or *E. dispar*. |
| Study size | 10 | Explain how the study size was arrived at  Determined by number patients positive for *E. histolytica/dispar* through antigen detection and/or through microscopy confirmed by PCR |
| Quantitative variables | 11 | Explain how quantitative variables were handled in the analyses. If applicable, describe which groupings were chosen and why  Relevant clinical and laboratory data were extracted, de-identified and entered in a Microsoft Access 2010 database. Data mining was performed with the SAP Business Objects (SAP, USA) program. |
| Statistical methods | 12 | (*a*) Describe all statistical methods, including those used to control for confounding For Prediction of *E. histolytica* infection in *E. histolytica/dispar* positive individuals sensitivity, specificity and likelihood ratios (LRs) were calculated, using the PCR as reference diagnostic standard. The medical records of all travelers and migrants proven to be infected with *E. histolytica* during the study period were then reviewed. An equal number of files of patients with confirmed E*. dispar* intestinal infection were randomly chosen and analyzed for a case control comparison.  Statistical analyses were done with Epi-Info (CDC 2015). Dichotomic variables where compared with Fisher exact test, minimum significance p<0.05. |
|  |  | (*b*) Describe any methods used to examine subgroups and interactions  NA |
|  |  | (*c*) Explain how missing data were addressed  For the case control study prediction of presence of E. histolytica by clinical data, was done only for the patients for which clinical data were available in the patient file |
|  |  | (*d*) *Cohort study*—If applicable, explain how loss to follow-up was addressed  NA  *Case-control study*—If applicable, explain how matching of cases and controls was addressed  NA  *Cross-sectional study*—If applicable, describe analytical methods taking account of sampling strategy |
|  |  | (*e*) Describe any sensitivity analyses |

Continued on next page

| Results | | |
| --- | --- | --- |
| Participants | 13* | (a) Report numbers of individuals at each stage of study—eg numbers potentially eligible, examined for eligibility, confirmed eligible, included in the study, completing follow-up, and analysed  From May 2006 till March 2016 parasitological examination was performed on 40,638 stool samples. Of these 868 (2.1 %) were found positive for *Entamoeba histolytica/dispar* through antigen detection and/or through microscopy confirmed by PCR. After removing results of follow-up samples, *E. histolytica* was detected in 30/826 samples: 3.6% of all stool samples positive for *E. histolytica/dispar* and 0.07% of all examined stool samples. *E. dispar* was detected in 714 (86.4%) samples, neither *E. histolytica* nor *E. dispar* in 50, and PCR was technically not feasible in 16 because no fresh stool sample was received. No co-infections with *E. histolytica* and *E. dispar* were found.  Antigen detection was performed in 396 of the 744 samples with *E. histolytica* or E. *dispar* as confirmed by PCR. In 16 samples, the antigen test was positive, with negative PCR for *E. histolytica* or *E. dispar* and negative microscopy (or microscopy not done), while in 1 *E. histolytica* PCR-confirmed patient antigen testing was positive with negative microscopy.  In 5 immediately examined fresh stool (n=3) and rectal pus samples (n=2) hematophagous trophozoites were found, all of which were confirmed as *E. histolytica.* |
|  |  | (b) Give reasons for non-participation at each stage  See in part above: (a)  In order to exclude pathogenicity by coinfections, clinical predictors were evaluated only on mono-infections with *E. histolytica* or *E. dispar*. Four patients with *E. dispar* infection were co-infected with *Giardia intestinalis* (n= 2)*, Strongyloides stercoralis* (n= 1) or *Schistosoma mansoni* (n= 1). Eight *E. histolytica* patients were co-infected with one or two of the following: *Giardia intestinalis* (n= 5), *Trichuris trichiura* (n= 2)*,* *Ankylostoma duodenale* (n= 1)*,* *Schistosoma mansoni* (n= 1) *and/or Campylobacter* (n= 1). |
|  |  | (c) Consider use of a flow diagram |
| Descriptive data | 14* | (a) Give characteristics of study participants (eg demographic, clinical, social) and information on exposures and potential confounders *E. histolytica* cases (n=30) were evenly distributed throughout the study period with no cluster phenomenon. Mean age was 36.8 years (range 4-80 years) and 21 (70%) samples were from males (Table 1). Regions of most recent travels were Africa (20 cases, 69%), Asia (8 cases, 24%), Latin-America (1 case, 3%) and Europe (1 case, 3%).  Of the 30 randomly selected *E. dispar* cases, mean age was 43 years (range 23-72 years) and 24 (80%) were males. Most recent travel regions were Africa (18 cases, 60%), Asia (3 cases, 10%), Latin-America (1 case, 3%) and Europe (8 cases, 27%).  Gender, age and travel destination were not significantly different between the *E. histolytica* and *E. dispar* cases.  Table 1 Baseline comparison patients with E histolytica and controls with E dispar   |  | E. histolytica  (n=30) | E. dispar  (n=30) | Significance | | --- | --- | --- | --- | | Gender M/F | 21/9 | 24/6 | Ns | | Age (mean) | 36.8 | 42.8 | Ns | | Africa/all | 20/30 | 18/30 | Ns | |
|  |  | (b) Indicate number of participants with missing data for each variable of interest  There were 22 mono-infections with E. histolytica and 26 mono-infections with E. dispar. Missing data for blood in stool, mucus in stool, cramps, watery diarrea, fever and any symptoms were 2, 4, 4, 3, 2 and 1 respectively for E. histolytica and 4, 4, 5, 4, 4 and 4 for E. dispar infected patients. |
|  |  | (c) *Cohort study*—Summarise follow-up time (eg, average and total amount) NA |
| Outcome data | 15* | *Cohort study*—Report numbers of outcome events or summary measures over time NA |
|  |  | *Case-control study—*Report numbers in each exposure category, or summary measures of exposure NA |
|  |  | *Cross-sectional study—*Report numbers of outcome events or summary measures |
| Main results | 16 | (*a*) Give unadjusted estimates and, if applicable, confounder-adjusted estimates and their precision (eg, 95% confidence interval). Make clear which confounders were adjusted for and why they were included  Overall, sensitivity of the different symptoms was low (Table 2). About 40% of *E. histolytica* infections were fully asymptomatic. Bloody stools, mucus and abdominal cramps were significantly correlated with *E. histolytica*, with a specificity of resp. 100, 100 and 90%. The presence of “any symptom” was not predictive for *E. histolytica* infection.   |  | ***E. histolytica*** | ***E. dispar*** |  |  |  |  | | | --- | --- | --- | --- | --- | --- | --- | --- | |  | **N/total** | **N/total** | **Sensitivity** | **Specificity** | **LR+** | **LR-** | **P-value** | | **Blood in stool** | 6/20 | 0/22 | 30 | 100 | 300* | 0.7 | 0.007 | | **Mucus in stool** | 4/18 | 0/22 | 22 | 100 | 222* | 0.8 | 0.030 | | **Cramps** | 8/18 | 2/21 | 44 | 90 | 4.7 | 0.6 | 0.009 | | **Watery diarrhea** | 6/19 | 11/22 | 32 | 50 | 0.6 | 1.4 | 0.2 | | **Fever** | 2/20 | 3/22 | 10 | 86 | 0.7 | 1.0 | 0.6 | | **Any Symptom** | 13/21 | 12/22 | 62 | 45 | 1.1 | 0.8 | 0.420 | |
|  |  | When only examination of direct smears was considered, the finding of trophozoites was predictive of E. histolytica (p=0.02), although sensitivity was very low (14%) (Table 3)  Tabel 3 Crosstab of finding of trophozoites for identification of E histolytica in direct smears   \|  \| E. histolytica \| \| E. dispar \| \|  \| LR \| \| --- \| --- \| --- \| --- \| --- \| --- \| --- \| \| Trophozoite \| 3 \|  \|  \| 17 \| 20 \|  \| \|  \|  \| 14% \| 3% \|  \|  \| 4.82 \| \|  \|  \| 86% \| 97% \|  \|  \| 0.88 \| \| Cyst \| 18 \|  \|  \| 557 \| 575 \|  \| \| Total \| 21 \|  \|  \| 574 \| 595 \|  \|   In contrast, the finding of trophozoites in fixed samples was not predictive of E. histolytica (p=0.2; Table 4).  Tabel 4 Crosstab of finding of trophozoites for identification of E histolytica in fixed samples   \|  \| E. histolytica \| \| E. dispar \| \|  \| LR \| \| --- \| --- \| --- \| --- \| --- \| --- \| --- \| \| Trophozoite \| 14 \|  \|  \| 250 \| 264 \|  \| \|  \|  \| 74% \| 63% \|  \|  \| 1.17 \| \|  \|  \| 26% \| 37% \|  \|  \| 0.71 \| \| Cyst \| 5 \|  \|  \| 146 \| 151 \|  \| \| Total \| 19 \|  \|  \| 396 \| 415 \|  \|   In 5 immediately examined fresh stool (n=3) and rectal pus samples (n=2) hematophagous trophozoites were found, all of which were confirmed as *E. histolytica.* |
|  |  | (*b*) Report category boundaries when continuous variables were categorized |
|  |  | (*c*) If relevant, consider translating estimates of relative risk into absolute risk for a meaningful time period |
| Other analyses | 17 | Report other analyses done—eg analyses of subgroups and interactions, and sensitivity analyses |
| Discussion | | |
| Key results | 18 | Summarise key results with reference to study objectives  In our Belgian reference clinic for tropical medicine we identified 3.6% (30/826) of *Entamoeba histolytica/dispar* infections as true *E. histolytica* infections by PCR.  In our study, the presence of blood or mucus in stool were highly predictive of true *E. histolytica* infections in case *Entamoeba histolytica/dispar* cysts or trophozoites were found on microscopy (LR+ > 100). Indeed we observed that none of the matched patients with *E. dispar* presented such symptoms. The presence of abdominal cramps had a LR+ of almost 5, which is also of good confirming value.  Finding trophozoites in direct smears had a LR+ for *E. histolytica* of 4.8, corresponding to a good confirming power. However, the LR- of 0.9 indicated that the absence of trophozoites, did not rule out *E. histolytica* infection. The non-significant LR+ of 1.2 for trophozoites in SAF fixed stool samples confirmed that this method cannot be used for species prediction.  Hematophagy was demonstrated in this study in which 5/5 hematophagous trophozoites found in immediately examined samples proved to be *E. histolytica*. |
| Limitations | 19 | Discuss limitations of the study, taking into account sources of potential bias or imprecision. Discuss both direction and magnitude of any potential bias  Our study has several limitations. It was a single-center study and the total number of *E. histolytica* infections found might not be representative for all returning travelers. In patients consulting at our center, we found 30 *E. histolytica* infections over 10 years, whereas the total number of *E. histolytica* infections diagnosed in our laboratory receiving stool samples from all over Belgium was 124 over the same period. Next, it was a retrospective study meaning that collection of data was not systematic. However, given the low number of confirmed *E. histolytica* infections in the 810 samples tested by PCR, the impact of missing analyses is likely marginal. In 50 samples positive by microscopy PCR was negative for both *E. histolytica* and *E. dispar* which probably indicates incorrect identification as infections with species such as *E. moshkovskii and E. polecki* are considered to be rare. A difference in clinical presentation in patients with *E. histolytica* and *E. dispar* infection is a possible confounding factor since clinicians might have asked less stool samples in asymptomatic patients. This might have underestimated the true prevalence of these infections. Nevertheless, the proportion of asymptomatic patients in our case-control group did not differ significantly. Furthermore, requesting stool analysis including antigen testing was clinician driven and an unknown number of *E. histolytica/dispar* infections may have been missed, in particular in asymptomatic travelers. The most trustworthy method to detect all *E. histolytica* and *E. dispar* infections, would have been to perform PCR on all stool samples of all symptomatic and asymptomatic travelers^7,27^. During the study period, this method was not part of common practice, though this may change with the deployment of multiplex PCR platforms to analyze stool samples. |
| Interpretation | 20 | Give a cautious overall interpretation of results considering objectives, limitations, multiplicity of analyses, results from similar studies, and other relevant evidence  In our Belgian reference clinic for tropical medicine we identified 3.6% (30/826) of *Entamoeba histolytica/dispar* infections as true *E. histolytica* infections by PCR. **This confirms the finding in other studies^21,22^ that the bulk of *Entamoeba histolytica/dispar* infections are caused by *E. dispar* amoeba.** True *E. histolytica* enteritis is a rare finding in patients presenting in our reference center, with on average less than 3 cases detected per year.  In a context where only microscopy is available, a patient presenting with blood or mucus in stool or cramps should anyhow be treated as amoebiasis if *Entamoeba histolytica/dispar* cysts/trophozoites are found. Nevertheless it is worth noting that a sizeable proportion of *E. histolytica* cases were asymptomatic. Relying only on one of the three clinical predictors would have missed 10 true *E. histolytica* infections in our cohort.  Finding trophozoites in direct smears had a LR+ for *E. histolytica* of 4.8, corresponding to a good confirming power. However, the LR- of 0.9 indicated that the absence of trophozoites, did not rule out *E. histolytica* infection. The non-significant LR+ of 1.2 for trophozoites in SAF fixed stool samples confirmed that this method cannot be used for species prediction.  The non-pathogenicity of *Entamoeba dispar* is questioned by several authors^26^,^15^. A study by Ximénez and colleagues suggests the existence of several different genotypes of *E. dispar* that can be associated to, or be potentiality responsible for, intestinal or liver tissue damage, similar to that observed with *E. histolytica*^14^. The difference in percentage of patients presenting with any symptom in patients with mono-infections with *E. histolytica* vs *E. dispar* was not significant (61% vs 55%, p value 0.42). This is not equivalent to stating that all symptoms of the 55% patients with symptomatic *E. dispar* infections were attributable to the *E. dispar* amoebae. Our study was not designed to show a pathogenic effect of *E. dispar*. However, the high frequency of symptoms in patients with *E. dispar* mono-infection supports Ximénez’s hypothesis, but symptoms in *E. histolytica* infected patients were clearly more often suggestive of intestinal tissue invasion. |
| Generalisability | 21 | Discuss the generalisability (external validity) of the study results  It was a single-center study and the total number of *E. histolytica* infections found might not be representative for all returning travelers. In patients consulting at our center, we found 30 *E. histolytica* infections over 10 years, whereas the total number of *E. histolytica* infections diagnosed in our laboratory receiving stool samples from all over Belgium was 124 over the same period.  In a context where only microscopy is available, a patient presenting with blood or mucus in stool or cramps should anyhow be treated as amoebiasis if *Entamoeba histolytica/dispar* cysts/trophozoites are found. Nevertheless it is worth noting that a sizeable proportion of *E. histolytica* cases were asymptomatic. |
| Other information | | |
| Funding | 22 | Give the source of funding and the role of the funders for the present study and, if applicable, for the original study on which the present article is based  NA. There were no funding sources for this study. |

*Give information separately for cases and controls in case-control studies and, if applicable, for exposed and unexposed groups in cohort and cross-sectional studies.

**Note:** An Explanation and Elaboration article discusses each checklist item and gives methodological background and published examples of transparent reporting. The STROBE checklist is best used in conjunction with this article (freely available on the Web sites of PLoS Medicine at http://www.plosmedicine.org/, Annals of Internal Medicine at http://www.annals.org/, and Epidemiology at http://www.epidem.com/). Information on the STROBE Initiative is available at www.strobe-statement.org.
